# Anti-Inflammatory Effects of Progesterone in Lipopolysaccharide-Stimulated BV-2 Microglia

**DOI:** 10.1371/journal.pone.0103969

**Published:** 2014-07-31

**Authors:** Beilei Lei, Brian Mace, Hana N. Dawson, David S. Warner, Daniel T. Laskowitz, Michael L. James

**Affiliations:** 1 Multidisciplinary Neuroprotection Laboratories, Duke University Medical Center, Durham, North Carolina, United States of America; 2 Department of Anesthesiology, Duke University Medical Center, Durham, North Carolina, United States of America; 3 Department of Neurology, Duke University Medical Center, Durham, North Carolina, United States of America; 4 Department of Neurobiology, Duke University Medical Center, Durham, North Carolina, United States of America; National Institute of Allergy and Infectious Diseases - Rocky Mountain Laboratories, United States of America

## Abstract

Female sex is associated with improved outcome in experimental brain injury models, such as traumatic brain injury, ischemic stroke, and intracerebral hemorrhage. This implies female gonadal steroids may be neuroprotective. A mechanism for this may involve modulation of post-injury neuroinflammation. As the resident immunomodulatory cells in central nervous system, microglia are activated during acute brain injury and produce inflammatory mediators which contribute to secondary injury including proinflammatory cytokines, and nitric oxide (NO) and prostaglandin E_2_ (PGE_2_), mediated by inducible NO synthase (iNOS) and cyclooxygenase-2 (COX-2), respectively. We hypothesized that female gonadal steroids reduce microglia mediated neuroinflammation. In this study, the progesterone’s effects on tumor necrosis factor alpha (TNF-α), iNOS, and COX-2 expression were investigated in lipopolysaccharide (LPS)-stimulated BV-2 microglia. Further, investigation included nuclear factor kappa B (NF-κB) and mitogen activated protein kinase (MAPK) pathways. LPS (30 ng/ml) upregulated TNF-α, iNOS, and COX-2 protein expression in BV-2 cells. Progesterone pretreatment attenuated LPS-stimulated TNF-α, iNOS, and COX-2 expression in a dose-dependent fashion. Progesterone suppressed LPS-induced NF-κB activation by decreasing inhibitory κBα and NF-κB p65 phosphorylation and p65 nuclear translocation. Progesterone decreased LPS-mediated phosphorylation of p38, c-Jun N-terminal kinase and extracellular regulated kinase MAPKs. These progesterone effects were inhibited by its antagonist mifepristone. In conclusion, progesterone exhibits pleiotropic anti-inflammatory effects in LPS-stimulated BV-2 microglia by down-regulating proinflammatory mediators corresponding to suppression of NF-κB and MAPK activation. This suggests progesterone may be used as a potential neurotherapeutic to treat inflammatory components of acute brain injury.

## Introduction

Numerous studies indicate that progesterone regulates multiple non-reproductive functions in the brain including cognition, memory, and neurogenesis [1], [2], [3], [4]. Progesterone elicits its effects via progesterone receptors (PRs), which include classical nuclear PRs (two major isoforms PR-A and PR-B) and recently recognized membrane PRs [3], [5], [6], [7]. The classical mechanism of progesterone action is mediated by nuclear PRs, which function as transcription factors by binding to specific progesterone response elements within the promoter region of target genes to modulate transcription and genomic networks [3], [4], [8]. Non-classical mechanisms have been recently suggested to involve membrane PRs and cytoplasmic kinase activation and signal cascades [3], [4], [8].

Progesterone exerts neuroprotective effects in several experimental acute brain injury models, including traumatic brain injury [9], [10], [11], [12], [13], [14], ischemic stroke [15], [16], [17], [18], [19], [20], [21], and subarachnoid hemorrhage [22], [23]. Progesterone has also demonstrated potential for clinical translation, having shown promise for treatment of traumatic brain injury in two independent phase II clinical trials [24], [25]; this approach is currently in phase III clinical trials (http://clinicaltrials.gov/ct2/show/record/NCT00822900). Preclinical studies suggest that progesterone may improve neurobehavioral outcomes by inhibition of neuroinflammation, oxidative stress, and neuronal death [10], [11], [13], [20]. However, specific underlying mechanisms remain unclear.

Microglia, the resident immune cells in the central nervous system, play important roles in the brain’s innate immunity and response to injury. Increasing evidence indicates microglial over-activation after acute brain injury results in excess production of proinflammatory mediators including tumor necrosis factor α (TNFα), prostaglandin E_2_ (PGE_2_), and nitric oxide (NO), which then contribute to secondary brain injury and exacerbate neuronal injury [26], [27], [28], [29]. NO and PGE_2_ are the products of inducible nitric oxide synthase (iNOS) and cyclooxygenase-2 (COX-2), respectively. Regulation of iNOS, COX-2 and TNFα expression involves transcriptional factor nuclear factor kappa B (NF-κB) [27], [30], [31] and mitogen activated protein kinases (MAPKs) [32], [33].

Lipopolysaccharide (LPS), a toll-like receptor ligand, induces microglia activation and neuroinflammation. The immortalized murine BV-2 microglia cell line is commonly used as a substitute for primary microglia in experimental studies [34], [35], [36]. To investigate progesterone’s molecular effects on neuroinflammation, BV-2 microglia cells were employed to test the effects of progesterone on the LPS-induced TNF-α, iNOS, and COX-2 expression. Since both NF-κB and MAPK pathways participate in the regulation of neuroinflammation [26], [27], [29], both pathways were examined as possible underlying molecular mechanisms.

## Materials and Methods

### Materials

Reagents and suppliers were: LPS (L6143), progesterone (P8783), mifepristone (M8046) (Sigma, St. Louis, MO); High Glucose Dulbecco’s Modified Eagle Medium (DMEM) (11995), phenol red free DMEM (31053), L-glutamine, sodium pyruvate, Pen/Strep and fetal bovine serum (Gibco, Grand Island, NY); NE-PER nuclear and cytoplasmic extraction reagents (78833), BCA protein assay kit (23227), restore stripping buffer (21059) and supersignal west dura extended duration substrate (34076) (Thermo Scientific, Rockford, IL); mouse TNF-α DuoSet enzyme-linked immunosorbent assay (ELISA**)** kit (DY410, R&D Systems, Minneapolis, MN); Antibodies against NF-κB p65 (4764), phospho-NF-κB (3033), IκB-α (9242), phospho-IκBα (9246), p38 (9212) and phospho-p38 (9211) MAPK, p44/42 (9102) and phspho-p44/42 (9101) MAPK, JNK (9252), phosphor-JNK (9251), GAPDH (2118) (Cell signaling, Beverly, MA); COX-2 antibody (160106) (Cayman chemical, Ann Arbor, MI); antibodies against progesterone receptor (sc-7208), NOS2 (sc-650) and secondary HRP antibodies (Santa Cruz Biotechnology, Santa Cruz, CA).

### Cell culture

BV-2, an immortalized mouse microglia cell line [34], was maintained in high glucose DMEM supplemented with 10% FBS and 1x penicillin/streptomycin in a humidified atmosphere containing 5% CO_2_ at 37°C.

### Immunofluorescence staining

Cells were grown on glass coverslips in complete medium for one day. After aspiration of medium, cells were fixed at room temperature for 10 min with 3.7% formaldehyde in PBS, permeablized in the same solution with 0.05% Triton X-100 for 10 min, and washed 3 times with PBS before 30 min of blocking in 10% FBS/PBS with 0.05% Triton X-100, and then incubated with primary antibodies to progesterone receptor (1∶100) overnight at 4°C in blocking buffer. After washing with PBS 3 times, cells were incubated at 25°C for 30 min with secondary goat anti-rabbit IgG Rhodamine Red X (Invitrogen) at 1∶1000 dilution in blocking buffer prior to washes with PBS, mounting with anti-fading buffer with DAPI and fluorescent microscopy analysis. Images were processed with Adobe Photoshop software (Adobe Systems Inc., San Jose, CA).

### Treatments

Prior to all experiments, BV-2 cells near confluence were trypsinized and plated in 12- or 6-well plates in complete DMEM for 1 day, and then washed and transferred to serum free and no phenol red DMEM with 1x L-glutamine and 1x sodium pyruvate for treatment. For time course experiments, cells were incubated with 30 ng/ml LPS for different time periods from 0 to 24 h. Pretreatment of escalated concentrations of progesterone (10^−8^ to 10^−6^ M) was given 1 h before addition of 30 ng/ml LPS. Post-treatment with progesterone was given 5, 30, or 60 min post LPS. In additional experiments, the progesterone receptor antagonist mifepristone (10^−9^ to 10^−7^ M) was given 30 min before progesterone. At pre-specified time points, cell supernatants were collected for TNF-α ELISA, and cells were lysed for western blot analyses.

### Quantification of TNF-α

Cell supernatants were collected and spun at 10,000 g for 1 min to exclude particulates. Supernatant TNF-α was measured by ELISA using a commercially available kit (R&D Systems) according to the manufacturer’s instruction.

### Whole cell lysate preparation

After aspiration of medium, cells were solubilized directly with addition of 2% SDS sample buffer to the plates. Cell lysates were transferred to new tubes, subjected to 15 seconds of sonication to shear DNA, and then stored at −80°C.

### Nuclear and cytoplasmic protein extraction

At various time points, cells were washed with ice-cold PBS, scraped off into PBS and spun down at 500 g for 2 min. Cell pellets were used for nuclear protein extraction using NE-PER Nuclear and Cytoplasmic Extraction Reagents (Thermo Scientific) according to manufacturer’s protocol. Protein concentrations were determined using a BCA protein assay kit (Thermo Scientific).

### Western blotting

Samples, loaded equally and resolved on 4–20% SDS polyacrylamide gels were transferred to polyvinylidene difluoride membranes. Membranes were blocked in TBS with 0.1% Tween-20 and 5% dry milk for 1 h at room temperature and then incubated with primary antibodies at 4°C overnight at ≥ 1/1000 dilutions. After incubation with horseradish peroxidase-conjugated secondary antibodies at 1/10,000 dilution for 1 h at room temperature, signal was detected using Western Dura Extended Duration Substrate and imaged with a HD2 CCD camera (Alpha Innotech, San Leandro, CA). When multiple probing was needed, membranes were stripped off immunoglobulin with restore stripping buffer before re-probing.

### Statistical analysis

Results are expressed as the mean ± standard deviation, compiled from *n* replicate experiments. Statistical significance was analyzed by one-way analysis of variance; correction for repeated measures was performed using Dunnett or Bonferroni post-tests where appropriate. All calculations were performed using GraphPad Prism (GraphPad Software, San Diego, CA) with *p*<0.05 considered significant.

## Results

### Localization of progesterone receptors in BV-2 cells

**Figure 1:**
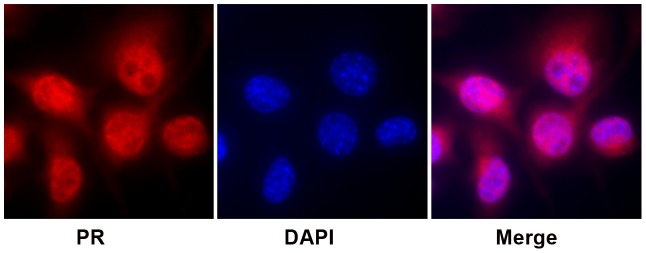
Localization of progesterone receptors in BV-2 microglia. Immunofluorescence staining detected the distribution of progesterone receptors in both nucleus and cytoplasm by some colocalization of receptors (PR, red) with nuclei (DAPI, blue).

To study the effects of progesterone in microglia, the expression of progesterone receptors was first examined. Utilizing immunofluorescence staining with antibody recognizing both PR-A and PR-B isoforms, under complete medium condition, co-localization of progesterone receptors (red) with nucleus (DAPI, blue) was observed, suggesting nuclear localization of progesterone receptors in BV-2 cells (Figure 1). Cytoplasmic distribution of PRs (red) was also detected. Taken together, PRs are expressed in both the cytoplasm and nucleus of BV-2 cells.

### Progesterone attenuated LPS-mediated TNF-α production

**Figure 2:**
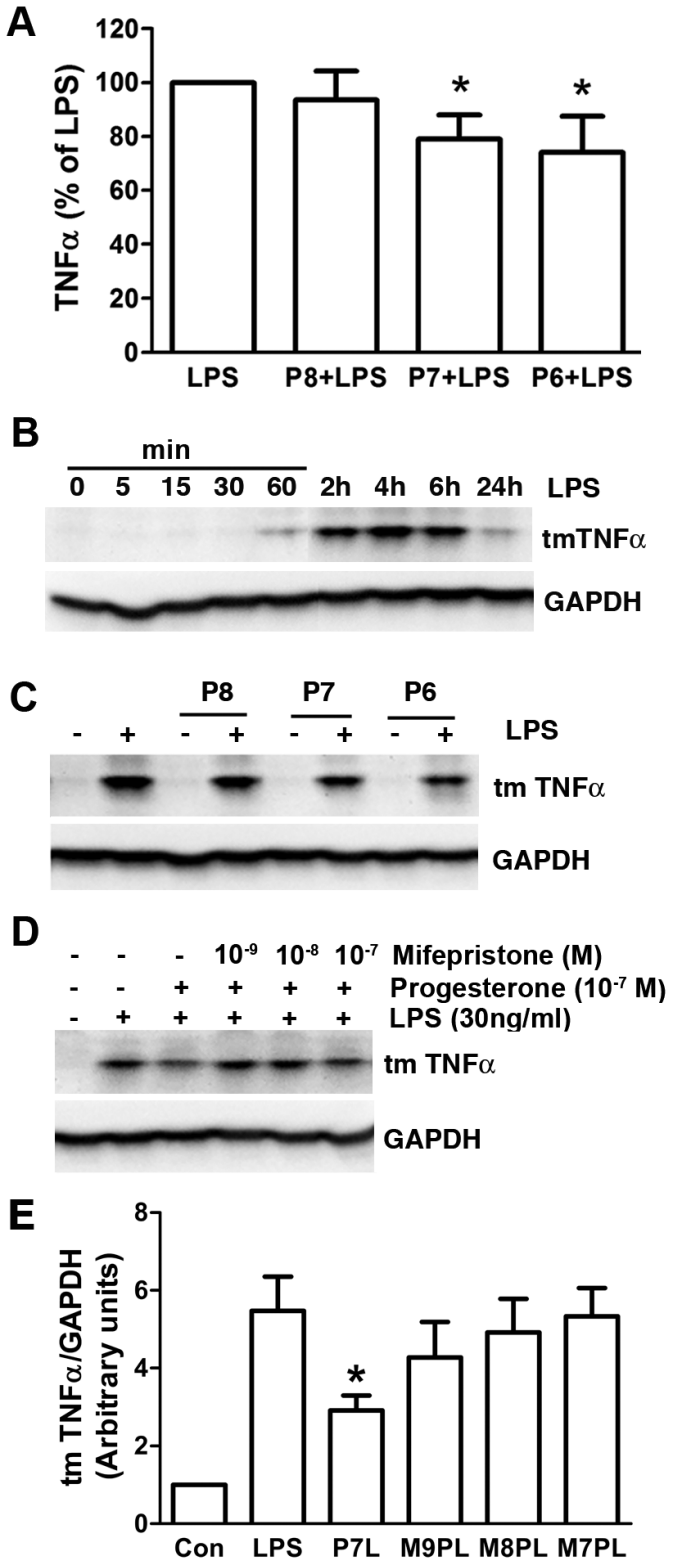
Progesterone attenuated LPS-stimulated TNF-α production. Cells were treated in serum-free DMEM. (A) Pretreatment of progesterone for 1 h dose-dependently suppressed LPS (30 ng/ml, 4 h)-mediated TNF-α secretion in cell supernants. *P<0.05 compared with LPS group, n = 7. P8, P7 and P6: progesterone at 10^−8^, 10^−7^ and 10^−6^ M. (B) The representative immunoblot demonstrated time course changes for transmembrane TNF-α (tmTNFα) and glyceraldehyde-3-phosphate dehydrogenase (GAPDH, a loading control) with LPS (30 ng/ml) stimulation from 0 to 24 h. (C) Pretreatment of progesterone for 1 h dose-dependently decreased LPS (30 ng/ml, 4 h)-stimulated tmTNF-α expression. The representative immunoblot (D) and summary of band densitometric quantifications (E) showed that progesterone receptor antagonist mifepristone reversed progesterone’s effect on tmTNF-α expression induced by 4 h LPS stimulation. *P<0.05 compared to LPS group, n = 3. P7L: progesterone 10^−7^ M+LPS; M9PL: mifepristone 10^−9^ M+progesterone 10^−7^ M+LPS; M8PL: mifepristone 10^−8^ M+progesterone 10^−7^ M+LPS; M7PL: mifepristone 10^−7^ M+progesterone 10^−7^ M+LPS.

To determine progesterone’s role in cytokine production, 4 h of LPS (30 ng/ml) exposure was used to stimulate BV-2 cell cytokine production in the presence of escalating doses of progesterone. Pretreatment of progesterone dose-dependently attenuated LPS-induced soluble TNF-α (sTNF-α) production in the supernatant. Significant sTNF-α decrease (approximately 20%) occurred with 10^−7^ and 10^−6^ M progesterone (Figure 2A). In time course experiments, as the precursor of sTNF-α, transmembrane TNF-α (mTNF-α) expression on BV-2 cells was found to increase at 1 h, reached maximum at 4 h, and then gradually decreased to 1-h levels by 24 h after LPS addition (Figure 2B). Progesterone also reduced LPS-induced mTNF-α expression in a dose dependent manner with similar maximal reduction at both 10^−7^ and 10^−6^ M (Figure 2C). Since 10^−7^ M is within the physiological concentration range of progesterone in blood in the female mouse [37], it was used for further experiments. Finally, mifepristone, a progesterone receptor antagonist, reversed progesterone’s effect on mTNF-α expression starting at 10^−9^ M (Figure 2D, 2E).

### Progesterone decreased LPS-induced iNOS and COX-2 expression

**Figure 3:**
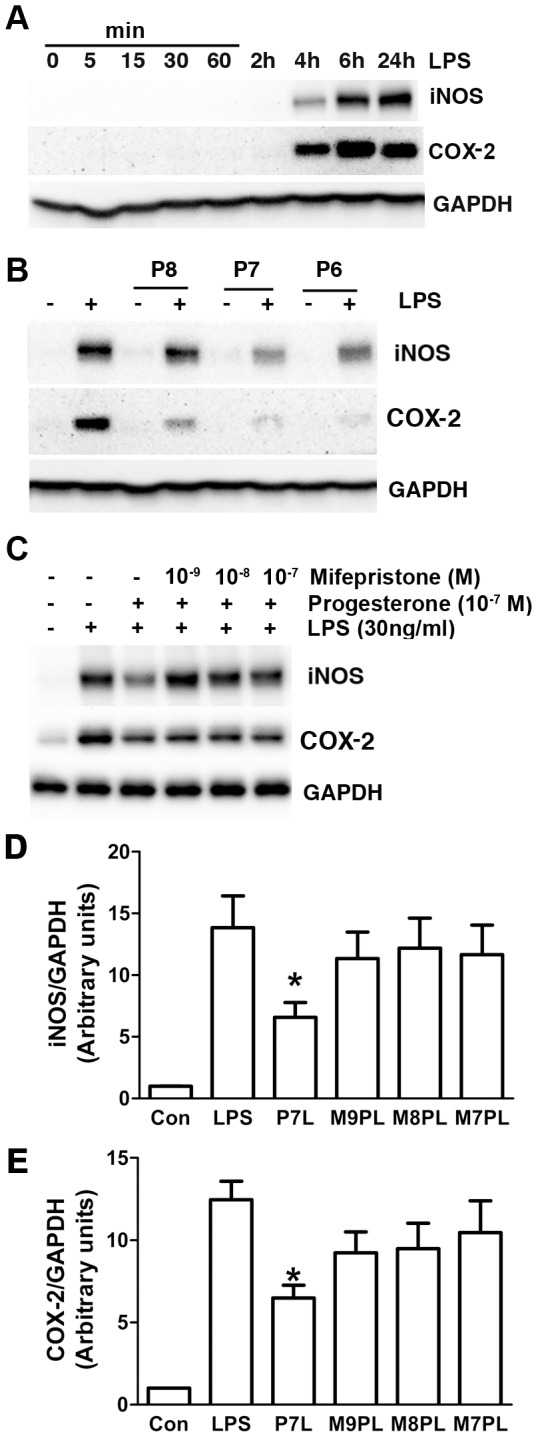
Progesterone decreased LPS-induced iNOS and COX-2 expression. Cells were treated in serum-free DMEM. (A) Representative immunoblots demonstrate time course changes for iNOS, COX-2 and GAPDH with LPS (30 ng/ml) stimulation. (B) Pretreatment of progesterone for 1 h dose-dependently decreased LPS (30 ng/ml, 4 h)-stimulated iNOS and COX-2 expression. P8, P7 and P6: progesterone at 10^−8^, 10^−7^ and 10^−6^ M. (C, D, E) Progesterone receptor antagonist mifepristone suppressed progesterone’s effect on LPS-induced iNOS and COX 2 expression for 20 h. *P<0.05 compared to LPS group, n = 3. P7L: progesterone 10^−7^ M+LPS; M9PL: mifepristone 10^−9^ M+progesterone 10^−7^ M+LPS; M8PL: mifepristone 10^−8^ M+progesterone 10^−7^ M+LPS; M7PL: mifepristone 10^−7^ M+progesterone 10^−7^ M+LPS.

To assess progesterone’s role in modulating production of NO and PGE2 in BV-2 cells, intracellular iNOS and COX-2 expression were measured. iNOS and COX-2 expression in BV-2 cells were increased beginning at 4 h, maximized at 6 h, and present through 24 h after LPS exposure (Figure 3A). Progesterone pretreatment decreased LPS-induced iNOS and COX-2 expression in a dose-dependent fashion, with maximal reduction at both 10^−7^ and 10^−6^ M (Figure 3B). Mifepristone inhibited progesterone’s effect (Figure 3C, D, E).

### Progesterone suppressed LPS-induced NF-κB activation

**Figure 4:**
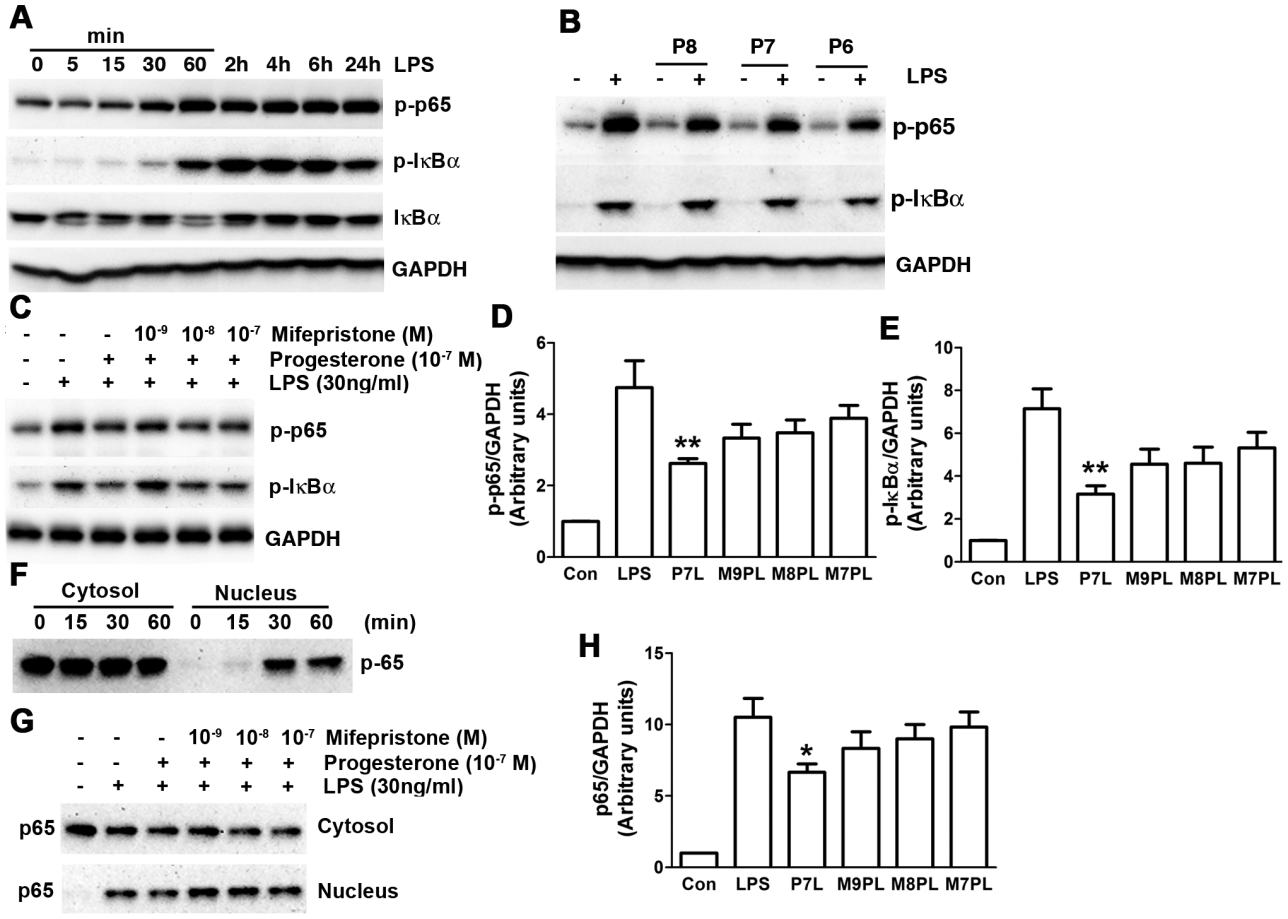
Progesterone suppressed LPS-induced NF-κB activation. Cells were treated in serum-free DMEM. (A) Representative immunoblots demonstrate time course changes for p-p65, p-IκBα, IκBα and GAPDH. (B) Progesterone pretreatment for 1 h dose-dependently decreased LPS (30 ng/ml, 4 h)-stimulated p65 and IκBα phosphorylation. P8, P7 and P6: progesterone at 10^−8^, 10^−7^ and 10^−6^ M. (C, D, E) Progesterone receptor antagonist mifepristone suppressed progesterone’s effect on p65 and IκBα phosphorylation. *P<0.05 compared to LPS group, n = 3. (F) LPS induced translocation of p65 from cytoplasm to nucleus. (G, H) Mifepristone reversed progesterone’s inhibition effect of p65 translocation by LPS for 1 hour. P7L: progesterone 10^−7^ M+LPS; M9PL: mifepristone 10^−9^ M+progesterone 10^−7^ M+LPS; M8PL: mifepristone 10^−8^ M+progesterone 10^−7^ M+LPS; M7PL: mifepristone 10^−7^ M+progesterone 10^−7^ M+LPS.

To assess progesterone’s role in modulating NF-κB activation in BV-2 cells, phosphorylation of inhibitory κBα (IκBα) and p65 and nuclear translocation of p65 were measured. IκBα and p65 phosphorylation was increased starting at 5 and 15 min respectively, reached maximum at 1–2 h, and was present through 24 h after LPS addition (Figure 4A). IκBα was decreased starting at 5 min, reached maximal reduction at 1 h, and started returning to basal level 2 h after LPS exposure. This indicates LPS induced IκBα degradation (Figure 4A). p65 translocation from the cytoplasm to the nucleus occurred by 15 min and reached maximal effect at 30 min of LPS exposure (Figure 4F). Progesterone pretreatment decreased IκBα and p65 phosphorylation (Figure 4B) and p65 translocation from cytoplasm to nucleus (Figure 4G, H), indicating progesterone suppresses NF-κB activation. Moreover, mifepristone suppressed progesterone’s inhibition of p65, IκBα phosphorylation (Figure 4C, D, E), and nuclear translocation (Figure 4G, H).

### Progesterone decreased LPS-mediated p38, JNK, and ERK MAPKs phosphorylation

**Figure 5:**
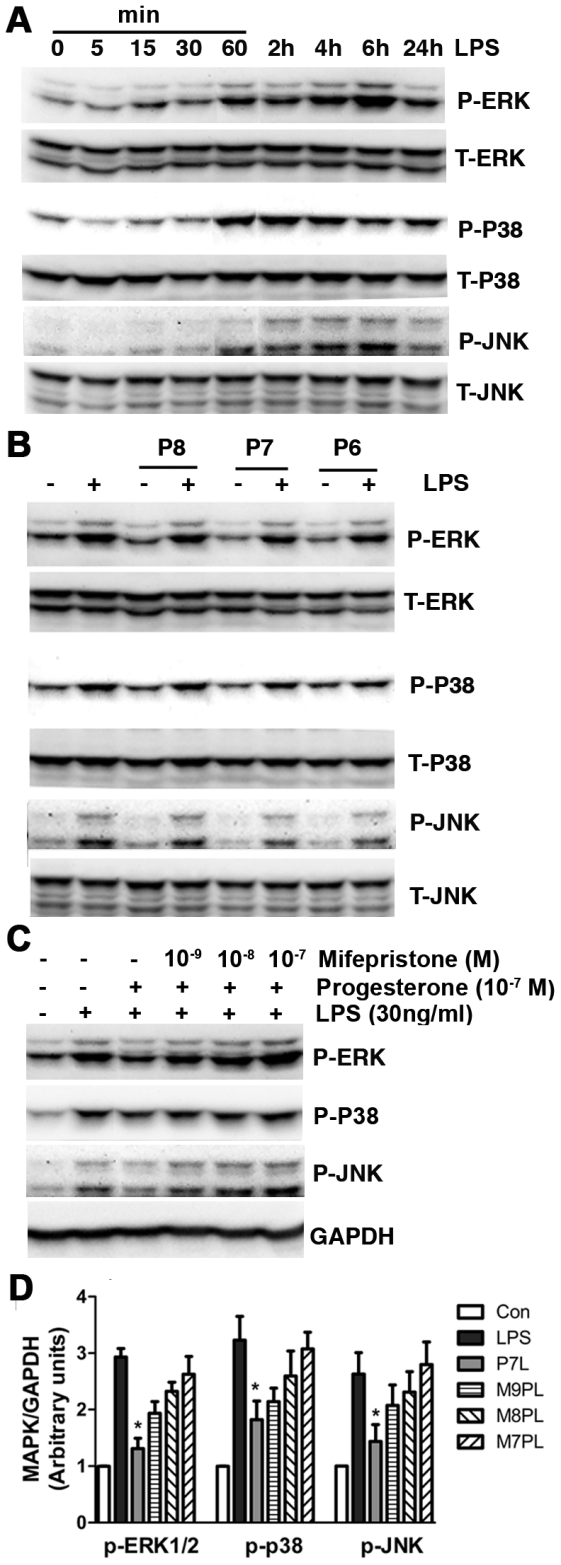
Progesterone reduced LPS-mediated phosphorylation of P38, JNK and ERK MAPKs. Cells were treated in serum-free DMEM. (A) Representative immunoblots demonstrate time course changes for p38, JNK and ERK MAPKs phosphorylation with LPS (30 ng/ml) stimulation. (B) Progesterone Pretreatment for 1 h dose-dependently decreased LPS (30 ng/ml, 4 h)-induced p38, JNK and ERK phosphorylation. P8, P7 and P6: progesterone at 10^−8^, 10^−7^ and 10^−6^ M. (C, D) Progesterone receptor antagonist mifepristone reversed progesterone’s effect on MAPK phosphorylation. *P<0.05 compared to LPS group, n = 3. P7L: progesterone 10^−7^ M+LPS; M9PL: mifepristone 10^−9^ M+progesterone 10^−7^ M+LPS; M8PL: mifepristone 10^−8^ M+progesterone 10^−7^ M+LPS; M7PL: mifepristone 10^−7^ M+progesterone 10^−7^ M+LPS.

To assess progesterone’s role in modulating MAPK activation in BV-2 cells, phosphorylation of p38, JNK, and ERK was measured. Time course experiments showed that phosphorylation of p38 was increased at 1 h, reached maximum at 2 h, and was present through 24 h after LPS addition, never returning to basal level (Figure 5A). JNK and ERK phosphorylation was increased beginning at 1 h, reached maximum at 4 h, and was present through 24 h after LPS exposure (Figure 5A). Therefore, 4 h stimulation of LPS was chosen for further MAPK studies. Progesterone pretreatment dose-dependently reduced LPS-stimulated p38, JNK, and ERK phosphorylation (Figure 5B). Mifepristone reversed progesterone’s effects (Figure 5C, D).

### Post-treatment progesterone did not alter LPS-induced inflammation

**Figure 6:**
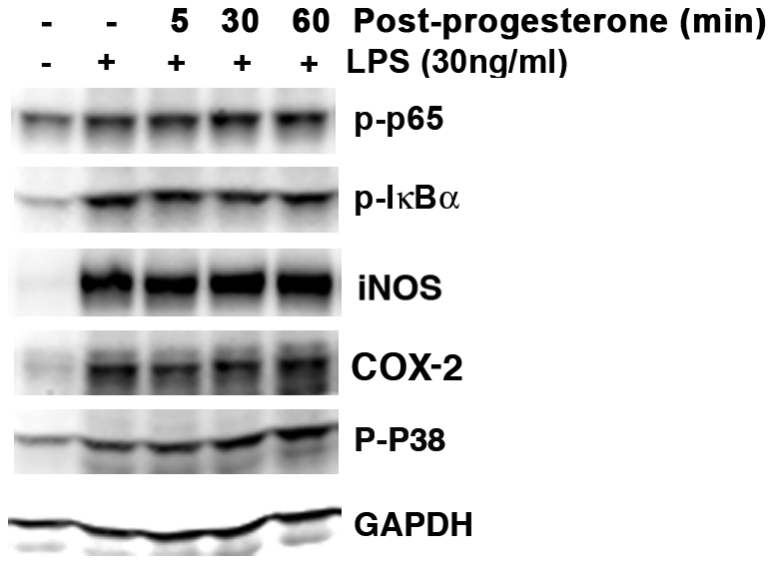
Post-treatment progesterone did not affect LPS-induced inflammation in BV-2 cells. Cells were treated in serum-free DMEM. Progesterone at 10^−7^ M was given 5, 30 or 60 min after LPS (30 ng/ml) addition. N = 3.

The effects of post-treatment progesterone were examined in LPS-stimulated BV-2 cells. P-p65, p-IκBα, iNOS, COX-2 and p-p38 expression were not affected by post-treatment dose (10^−7^ M) of progesterone given 5, 30 or 60 min post-LPS stimulation (Figure 6).

## Discussion

In this study, progesterone’s effects on TNF-α, iNOS, and COX-2 expression in LPS-stimulated BV-2 microglia were investigated as possible molecular mechanisms for the downregulation of post-injury neuroinflammatory responses mediated by NF-κB and MAPK pathways. LPS upregulated TNF-α, iNOS, and COX-2 expression in BV-2 cells. Progesterone attenuated LPS-stimulated TNF-α, iNOS, and COX-2 expression in a dose-dependent fashion. In addition, progesterone suppressed LPS-induced NF-κB activation by decreasing IκBα phosphorylation, p65 phosphorylation, and p65 nuclear translocation. Moreover, progesterone reduced LPS-mediated phosphorylation of p38, JNK, and ERK MAPKs in BV-2 cells. The progesterone antagonist mifepristone antagonized these effects.

Inflammation is a key factor of secondary brain damage. TNF-α, a potent proinflammatory cytokine, is upregulated immediately after acute brain injury [38], [39]. mTNF-α can act as a ligand to type 1 and 2 TNF-α receptors of target cells and mediates cytotoxic activity. Thus, mTNFα is involved in local inflammatory responses [40], [41]. sTNF-α is cleaved from its precursor, mTNF-α, by TNF-α-converting enzyme and released to remote tissue. In this study, progesterone pretreatment decreased not only sTNF-α production, but also mTNF-α expression, indicating inhibition by progesterone was upstream of mTNFα formation. Further, progesterone suppressed the biological activities mediated by both sTNF-α and mTNF-α in BV-2 cells, such as neuroinflammation and neuronal damage.

It has been extensively reported that NF-κB plays a critical role in regulation of inflammatory responses [26], [42], [43]. In mammals, five NF-κB subunits have been identified, including p50, p52, p65 (RelA), RelB and c-Rel [44]. NF-κB consists of dimers composed primarily of p65 and p50 subunits. Inactive NF-κB dimers are sequestered in the cytoplasm by association with IκB proteins. Typically, phosphorylation of IκB by IκB kinases targets it for ubiquitination and subsequent proteasomal degradation. This leads to release and translocation of NF-κB dimers to the nucleus. In the nucleus, NF-κB binds to the specific DNA sequence in the promoter region of target genes and modulates their transcription. The genes of TNF-α, iNOS, and COX-2 are known transcription targets of NF-κB [31], [45].

In agreement with the involvement of NF-κB in regulating TNF-α, iNOS, and COX-2 gene transcription, mTNF-α expression was first observed at 1 h and iNOS and COX-2 expression was first observed at 4 h in BV-2 microglia after LPS addition, while the NF-κB pathway was activated as early as 15 min after LPS exposure. LPS dramatically induced IκB degradation from 5 min, and IκBα proteinwas almost undetectable at 1 h. NF-κB p65 nuclear translocation started at 15 min and reached maximum at 30 min after LPS exposure. Progesterone suppressed the LPS-induced p65 translocation, and its receptor antagonist mifepristone completely blocked the inhibition by progesterone, indicating NF-κB in the possible mechanisms by which progesterone decreases proinflammatory mediators. Progesterone has been reported to suppress NF-κB activity by increase IκBα transcription or inhibit p65 expression [46].

Aside from NF-κB, the MAPK pathway has also been reported to be involved in neuroinflammation, especially the p38 pathway [47], [48], [49], [50]. Compared to wild type mouse microglia, primary microglia cultures from p38α MAPK knockout mice showed a diminished proinflammatory cytokine response to LPS, suggesting that the p38α MAPK pathway is a key regulator of proinflammatory cytokine overproduction [33]. In our study, p38, ERK, and JNK phosphorylation was first observed 1 h after LPS addition, progesterone attenuated this phosphorylation, and the antagonist mifepristone reversed progesterone’s effect, implicating the possible involvement of MAPK pathways in the microglial modulatory effects of progesterone.

Interestingly, these effects of progesterone were lost when administered after LPS-stimulation. Since microglia activation after acute brain injury is a relatively gradual process, which starts as early as a couple of hours and peaks at 3 to 5 days after ischemic stroke or intracerebral hemorrhage [51], the present finding suggests that progesterone’s therapeutic window should be chosen before the peak time of microglia activation.

Although the BV-2 cell line has been most commonly used in microglia study and evaluated to be a valid substitute for primary microglia in many conditions, clear differences between BV-2 and primary microglia was also observed such as stronger reaction to LPS and a much larger number of genes regulated significantly by LPS in primary microglia [35]. Even primary neonatal microglia were reported to function differently from adult microglia [52], suggesting that primary adult microglia might be more appropriate for *in vitro* study of aged CNS diseases.

## Conclusion

In LPS-stimulated BV-2 microglia, progesterone exhibits anti-inflammatory effects by suppressing NF-κB and MAPK activation. This is associated with downregulation of proinflammatory mediator expression. Thus, progesterone may have a role as a potential neuroprotective agent to treat acute neuroinflammation, consistent with its reported efficacy in preclinical models of acute brain injury. Confirmation in primary adult microglial cultures will serve to strengthen this proposition.
